# Influence of cyanobacteria, mixotrophic flagellates, and virioplankton size fraction on transcription of microcystin synthesis genes in the toxic cyanobacterium *Microcystis aeruginosa*

**DOI:** 10.1002/mbo3.538

**Published:** 2017-09-25

**Authors:** Pia I. Scherer, Carolin Absmeier, Maria Urban, Uta Raeder, Juergen Geist, Katrin Zwirglmaier

**Affiliations:** ^1^ Aquatic Systems Biology Unit Department of Life Sciences Weihenstephan Limnological Research Station Iffeldorf Technical University of Munich Munich Germany; ^2^ Bundeswehr Institute of Microbiology Munich Germany

**Keywords:** *mcyB*, *mcyD*, *Microcystis aeruginosa*, *Ochromonas danica*, *Synechococcus elongatus*, toxic algal blooms

## Abstract

Toxic cyanobacteria such as *Microcystis aeruginosa* are a worldwide concern in freshwater reservoirs. Problems associated with their mass occurrence are predicted to increase in the future due to global warming. The hepatotoxic secondary metabolite microcystin is of particular concern in this context. This study aimed to determine whether co‐occurring microorganisms influence the expression of microcystin biosynthesis genes. To this end, we performed cocultivation experiments and measured *mcyB* and *mcyD* transcripts in *M. aeruginosa* using RT‐qPCR. We utilized representatives from three different plankton groups: the picocyanobacterium *Synechococcus elongatus*, the unicellular flagellate grazer *Ochromonas danica*, and virioplankton from two different lakes. The presence of *S. elongatus* significantly increased *mcyB* and *mcyD* transcription in *M. aeruginosa*. Cocultivation with the mixotrophic chrysophyte *O. danica* did not increase the transcription of *mcyB* and *mcyD*; in fact, *mcyD* transcripts decreased significantly. The virioplankton size fraction of environmental water samples induced a significant increase in *mcyB* and *mcyD* transcription when obtained from lakes with cyanobacterial blooms. Our results show that co‐occurring microorganisms influence the expression of microcystin biosynthesis genes in *M. aeruginosa*.

## 1 INTRODUCTION

Many strains of *Microcystis aeruginosa* and other cyanobacteria produce the toxic secondary metabolite microcystin, which inhibits protein phosphatases in eukaryotes (MacKintosh, Beattie, Klumpp, Cohen, & Codd, 1990) and is thus toxic to humans and animals. Therefore, microcystin is a growing concern in freshwater management worldwide (Moreira, Spillane, Fathalli, Vasconcelos, & Antunes, 2014; Sivonen & Jones, 1999; Van Gremberghe et al., 2011).

The adverse effects of microcystin on aquatic organisms have been studied intensely. Particular attention has been given to mussels (Juhel et al., 2006), fish (Hou et al., 2016; Liu, Tang, Li, Hu, & Wang, 2015; Xie et al., 2015), members of the zooplankton community such as cladocerans (Dao, Do‐Hong, & Wiegand, 2010; DeMott, Zhang, & Carmichael, 1991; Fulton & Paerl, 1987b; Herrera, Echeverri, & Ferrão‐Filho, 2015; Ortiz‐Rodríguez, Dao, & Wiegand, 2012; Wiegand, Peuthert, Pflugmacher, & Carmeli, 2002), and aquatic macrophytes (Mitrovic, Allis, Furey, & James, 2005; Pflugmacher, 2002, 2004; Yin, Huang, Li, & Liu, 2005). However, much less is known about the inverse relationship, that is, the effect that co‐occurring organisms might have on microcystin producers such as *Microcystis* sp. One of the few studies to address this question found an increase in McyB protein in *Microcystis* sp. caused by the presence of the dinoflagellate *Peridinium gatunense* (Vardi et al., 2002). The little data available on *M. aeruginosa* toxin gene expression are limited to cocultivation with *Daphnia magna*, a metazoan grazer, which was observed to cause overexpression of *mcyA* in *M. aeruginosa* (Pineda‐Mendoza, Zúñiga, & Martínez‐Jerónimo, 2014). In addition, colony formation as a response to grazing danger has been observed but is not restricted to toxic strains (Fulton & Paerl, 1987a; Yang, Kong, Shi, & Cao, 2006).

The fate of *M. aeruginosa* cells in the environment is affected by co‐occurring microorganisms in several ways: Other phototrophs compete with *M. aeruginosa* for similar resources. At the same time, *M. aeruginosa* may be subject to and cause of interactions by means of secondary metabolites. Furthermore, *M. aeruginosa* cells are grazed on by zooplankton or utilized as a host by intracellular parasites such as bacteriophages, which can cause lysis of the *M. aeruginosa* cell.

In aquatic environments, several strains and species of cyanobacteria generally co‐occur (Al‐Tebrineh et al., 2012; Glowacka, Szefel‐Markowska, Waleron, Lojkowska, & Waleron, 2011; Via‐Ordorika et al., 2004; Zwart et al., 2005), and studies suggest that microcystin producers may have an advantage in the competition for micronutrients such as iron (Lukač & Aegerter, 1993; Utkilen & Gjølme, 1995). Interaction of cyanobacteria with other members of the microbial community by means of secondary metabolites has been described and is an ongoing and expanding field of study (Kaplan, Weiss, & Sukenik, 2016; Kaplan et al., 2012). The picocyanobacterium *Synechococcus elongatus* is of particular interest in this context because it is almost ubiquitously distributed (Stockner, Callieri, & Cronberg, 2002). The effect of *Synechococcus* on *Microcystis* gene expression is of ecological relevance since those cyanobacteria do not only co‐occur (Fortin et al., 2015; Kolmonen, Sivonen, Rapala, & Haukka, 2004; Magana‐Arachchi, Wanigatunge, & Liyanage, 2011; Ouellette, Handy, & Wilhelm, 2006; Zwart et al., 2005), but also can constitute the two most dominant cyanobacteria species in the phytoplankton community (Berry et al., 2016; Feng et al., 2016; Teneva, Mladenov, Belkinova, Dimitrova‐Dyulgerova, & Dzhambazov, 2010; Ye et al., 2011).

Cyanobacteria serve as prey for unicellular zooplankton. For instance, protists such as the mixotrophic flagellate *Ochromonas* sp. have been shown to feed on *Microcystis* sp. in the laboratory (Wilken, Wiezer, Huisman, & Van Donk, 2010). Furthermore, environmental studies have revealed that *Ochromonas* spp. co‐occur with *Microcystis* spp. in natural habitats (Van Donk et al., 2009) and that *Ochromonas* spp. are among the most widespread and abundant bacterivores in aquatic environments (Arndt et al., 2000). For these reasons, Wilken, Verspagen, Naus‐Wiezer, Van Donk, and Huisman (2014) evaluated *Ochromonas* as a tool to control *Microcystis* blooms.

Besides being grazed on by protists, lysis by bacteriophages is a major mortality factor for cyanobacteria (Proctor & Fuhrman, 1990). Viruses are the most abundant biological entities in both marine and freshwater ecosystems (Bergh, Børsheim, Bratbak, & Heldal, 1989; Wommack & Colwell, 2000). Laboratory and field studies suggest that phages regulate bloom dynamics of *Microcystis* sp. (Manage, Kawabata, & Nakano, 1999; Tucker & Pollard, 2005; Yoshida et al., 2008). Cyanophages that infect *M. aeruginosa* have been isolated in earlier studies and have been discussed as a tool for biological control of toxic cyanobacteria (Phlips, Monegue, & Aldridge, 1990; Yoshida et al., 2006).

The biological function of microcystin is still under investigation and while its role in the survival of the *Microcystis* cell is not yet fully understood, there have been several foci of research over the years: Microcystin, a peptide toxic to many animals, was suspected to act as a defense against grazers such as *Daphnia* (Kurmayer & Jüttner, 1999; Pineda‐Mendoza et al., 2014). Other studies, however, provide phylogenetic evidence (Rantala et al., 2004) as well as proteomic and physiological data (Zilliges et al., 2011) that suggest that a defense against metazoan grazers is not microcystin's primary function. Nevertheless, those grazers might have an effect on the expression of microcystin biosynthesis genes (Pineda‐Mendoza et al., 2014). In addition, some doubt has been cast on microcystin as a primary defense mechanism against unicellular grazers such as mixotrophic flagellates (Wilken et al., 2010), albeit the situation is less clear here. Instead, more recent studies have focused increasingly on the intracellular and regulatory functions of microcystin within *Microcystis* cells (Makower et al., 2015; Meissner, Fastner, & Dittmann, 2013; Zilliges et al., 2011).

However, the discoveries that microcystin has multiple intracellular effects and that defense against metazoan grazers is not its primary role do not rule out the possibility that microcystin regulation might be affected by secondary metabolites of other microbial community members (viruses, protists, or other cyanobacteria). This is suggested by observations that toxins may increase in the presence of other microorganisms (Vardi et al., 2002). For this reason, it is of the utmost importance to understand how interaction between members of the microsphere and toxigenic cyanobacteria affects their expression of toxicity genes. In addition, any biological agent considered for use in the control of harmful algal blooms should first be scrutinized for its potential effects on toxin gene expression to prevent exacerbation of the problem.

The effect of co‐occurring microorganisms on toxin gene expression in *Microcystis* sp. is not well understood. The aim of this study was to determine whether transcription of the microcystin biosynthesis genes *mcyB* and *mcyD* is altered by the presence of different types of microorganisms. We cocultivated *M. aeruginosa* with representative protists, cyanobacteria, or the virioplankton size fraction of environmental water samples from two different lakes and assessed the transcription of *M. aeruginosa* microcystin biosynthesis genes.

## 2 EXPERIMENTAL PROCEDURES

### 2.1 Sampling and preparation of environmental samples

Water samples for experiments with the virioplankton size fraction were collected from Lake Klostersee and Lake Bergknappweiher, two Bavarian lakes frequently presenting with cyanobacterial blooms. Lake Klostersee (GPS coordinates 48.08, 11.96) is a polytrophic artificial lake with a maximum water depth of 2.5 m, located 545 m above sea level in Ebersberg, Germany (Gesundheitsamt Landratsamt Ebersberg, 2014). Lake Bergknappweiher (GPS coordinates 47.85, 11.23) is a small meso‐eutrophic lake with a maximum water depth of 2.5 m, located 617 m above sea level, and about 50 km south‐west of Munich, Germany (Teubner et al., 2004). Water samples (4 L) were collected on 25 June 2015 from Lake Klostersee and on 27 July 2015 and 27 August 2015 from Lake Bergknappweiher. Samples were taken from surface water near the shore, and bacterioplankton and zooplankton were removed by filtering the water through a 0.2‐μm pore‐size cellulose nitrate filter (Sartorius, Göttingen, Germany). The filtrate containing the virioplankton and other particles smaller than 0.2 μm as well as dissolved organic matter is referred to as 0.2 μm filtrate in this study. Subsequently, cross‐flow filtration was performed to concentrate the particles present in the 0.2 μm filtrate (Vivaflow50, 10 000 MWCO, Sartorius, Göttingen, Germany). We obtained 100 ml cross‐flow filtrate from each liter of 0.2 μm filtrate. This 10‐fold concentration of particles larger than 10 kDa is referred to as particle concentrate. The particle concentrate contains all substances present in the 0.2 μm filtrate and is enriched in particles such as viral particles. For some experiments 0.2 μm filtrate was inactivated by means of autoclaving (121°C, 20 min) and is referred to as autoclaved filtrate.

### 2.2 Cultivation and strains

The microcystin‐producing cyanobacterium *M. aeruginosa* SAG14.85 and the mixotrophic flagellate *Ochromonas danica* SAG933‐7 were obtained from the Culture Collection of Algae at Göttingen University in Germany (SAG). *Ochromonas* was selected for being a phytoplankton grazer. The picocyanobacterium *S. elongatus* PCC7942 was obtained from the Pasteur Culture Collection of Cyanobacteria at the Institut Pasteur in Paris, France (PCC). *Synechococcus* was selected due to its almost ubiquitous distribution and its ability to compete for similar resources as *Microcystis* (Stockner et al., 2002).

All three strains were cultured at 25°C and under a light–dark regime of 14 hr light and 10 hr dark. Cool daylight with an intensity of 100 μmol/s per m^2^ was provided by MASTER TL5 HO 39W/865 1SL fluorescent light tubes (Phillips, Amsterdam, Netherlands). Cultures were regularly checked for contamination via microscopy (LEICA DM R, Leica Microsystems, Wetzlar, Germany).

*Microcystis aeruginosa* and *S. elongatus* cells were grown separately for 10 ± 2 days in 100 ml volumes of BG‐11 medium (Rippka, Deruelles, Waterbury, Herdman, & Stanier, 1979) supplemented with 0.5 mmol/L ammonium chloride in 300 ml borosilicate Erlenmeyer flasks. Both strains grew unicellular and were precultured under the same experimental conditions. Optical density at 730 nm (OD_730_) was measured with a spectrophotometer (model 150‐20, Hitachi, Chiyoda, Japan) to monitor cyanobacterial growth. Growth rates for *M. aeruginosa* and *S. elongatus* without treatment were 0.15/day and 0.13/day, respectively. Spent medium from *S. elongatus* cell cultures was obtained by filtering cultures through a 0.2‐μm pore‐size cellulose nitrate filter (Sartorius, Göttingen, Germany) to remove any cells from the medium.

*Ochromonas danica* cells were grown in 50 ml Ochromonas medium (Röderer, 1986) in 100 ml borosilicate Erlenmeyer flasks. *Ochromonas danica* was grown for 5 days to a concentration of 1.5 ± 0.5 × 10^6^ cells/ml, and its growth was monitored by counting cells immobilized with 1% (v/v) glycerine in a Neubauer improved counting chamber (Paul‐Marienfeld GmbH & Co. KG, Lauda Königshofen, Germany) under a microscope (Leica DM R, Leica Microsystems, Wetzlar, Germany). *Ochromonas danica* cells grew unicellular and were precultured under the same conditions.

For each experimental condition, three replicates of *M. aeruginosa* culture were grown to the early exponential phase (OD_730_ of 0.5 ± 0.1). At this point, equal volumes of either control medium, autoclaved filtrate, 0.2 μm filtrate, particle concentrate, *S. elongatus* (grown to OD_730_ of 0.15 ± 0.01), or *S. elongatus* spent medium were added, or *O. danica* was added at the desired concentrations (10^3^ or 10^4^ cells/ml) to the *Microcystis* culture and incubated for 48 hr before harvesting. *Microcystis aeruginosa* OD_730_ values at the time of harvesting are shown in Table S2.

### 2.3 Plaque assay

Plaque assay was performed as described previously (Millard, 2009) with modifications to accommodate bacteriophages from a freshwater environment. In short, concentrated log phase *M. aeruginosa* cells were mixed with 0.2 μm filtrate and grown on 1% (w/v) agar plates with 0.4% (w/v) top agar. Plates were prepared with washed agar agar (Carl Roth, Karlsruhe, Germany) and BG‐11 medium (Rippka et al., 1979) supplemented with 0.5 mmol/L ammonium chloride.

### 2.4 Humic acid treatment

To mimic the effect of the humic acids present in Lake Bergknappweiher water under laboratory conditions, artificial humic acid extract (HuminFit, Dohse Aquaristik GmbH & Co. KG, Grafschaft, Germany) was used to obtain a medium of visual similar color to the Lake Bergknappweiher water. To this end, 80 μl artificial humic acid extract was added to 200 ml of *M. aeruginosa* culture (4 times the manufacturer's recommended concentration). To test a 2.5 times higher humic acid concentration, 200 μl artificial humic acid extract (10 times the manufacturer's recommended concentration) was added to 200 ml of *M. aeruginosa* culture. For absorption spectra see Figure S3.

### 2.5 Harvesting and RNA extraction

Harvesting of cell cultures and RNA extraction were carried out as described previously (Scherer, Raeder, Geist, & Zwirglmaier, 2017). Briefly, 20 ml liquid culture was filtered through a 0.2‐μm pore‐size cellulose nitrate filter (Sartorius, Göttingen, Germany). RNA from the filter was extracted as described in Penn, Wang, Fernando, and Thompson (2014), cleaned using the RNA Clean & Concentrator kit (Zymo Research, Irvine, USA), and quantified and quality checked using a NanoVue Plus spectrophotometer (GE healthcare, Little Chalfont, UK) and 1% agarose gel stained with GelRed (Biotium, Hayward, CA, USA), respectively.

### 2.6 RT‐qPCR

Relative quantification of transcripts by reverse‐transcriptase quantitative polymerase chain reaction (RT‐qPCR) and subsequent data analyses were performed as described previously (Scherer et al., 2017). Two target genes, *mcyB* and *mcyD*, representatives of the microcystin synthetase gene cluster, and four reference genes (*rpoC1*,* gltA*,* rpoD*, and *GAPDH*) were used in this assay. Suitability of the four reference genes as reference gene panel for each experimental condition was confirmed with geNorm (*M* < 0.5). For primers and cycling conditions refer to Scherer et al. (2017). Normalized expression was determined as described in Vandesompele et al. (2002). In order to determine relative normalized expression, expression level of the control samples is set to one.

### 2.7 Statistical analysis

To test for statistically significant changes in gene expression, fold‐change values were tested for normal distribution (Shapiro–Wilk test). Subsequently, the data were analyzed using one‐way ANOVA with the level of significance defined as *p* ≤ .05. If applicable, Tukey's post hoc test was performed. All statistical analyses were performed using PAST v3.10 software (Hammer, Harper, & Ryan, 2001).

## 3 RESULTS AND DISCUSSION

### 3.1 Response to cyanobacteria

**Figure 1:**
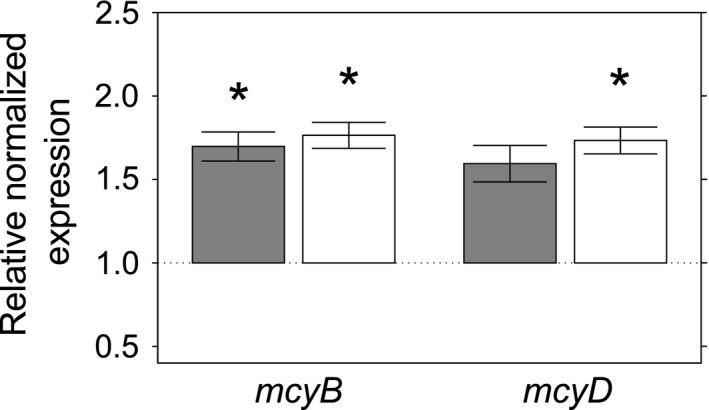
Relative normalized *mcyB* (left) and *mcyD* (right) mRNA expression in *Microcystis aeruginosa*. Error bars show standard error of the mean. *Statistically significant difference from control treatment, *p* ≤ .05. Cocultivation of *M. aeruginosa* with cell‐free spent medium from *Synechococcus elongatus* culture (gray) or *S. elongatus* cells in spent medium (white) for 48 hr. The control treatment, BG‐11 medium, was used for normalization

Cocultivation of *M. aeruginosa* with the picocyanobacterium *S. elongatus* or spent medium from *S. elongatus* cultures resulted in increased transcription of microcystin biosynthesis genes in *M. aeruginosa* (Figure 1). Transcription of *mcyB* significantly increased in the presence of spent medium (*p *= .003) or cells of *S. elongatus* (*p *= .001) compared to the control treatment. For *mcyD* transcripts, a significant increase was found for cultures exposed to *S. elongatus* cells (*p *= .025) but not cultures exposed to mere spent medium of *S. elongatus* cells (*p *= .064). These results indicate that microcystin biosynthesis genes in *M. aeruginosa* might be upregulated by a mechanism involving the direct presence of *S. elongatus* or, to a lesser extent, the presence of soluble compounds released into the medium by this cyanobacterium. Close communication of cyanobacteria with other members of the biosphere has been observed in several previous studies (Briand, Bormans, Gugger, Dorrestein, & Gerwick, 2016; Kaplan et al., 2012, 2016). One possible means of interaction between co‐occurring microorganisms is through small molecules. *Synechococcus elongatus*, for instance, has been shown to release a large variety of such small molecules into its surroundings (Fiore, Longnecker, Soule, & Kujawinski, 2015). However, little is known about how these molecules influence the expression of toxicity genes. The identification and characterization of the secondary metabolites or other chemicals that mediate the interaction between *S. elongatus* and *M. aeruginosa* are avenues for future research.

A possible allelopathic role of microcystin against *S. elongatus* would explain the observed upregulation of *mcyB* and *mcyD* in the presence of *S. elongatus*. Negative allelopathic effects of microcystin on picocyanobacteria have been described previously by Phelan and Downing (2014) and Hu, Liu, and Li (2004). In our study, however, growth curves failed to reveal any effect of spent medium from *M. aeruginosa* cultures on the growth of *S. elongatus* cultures (data not shown).

### 3.2 Response to mixotrophic flagellates

**Figure 2:**
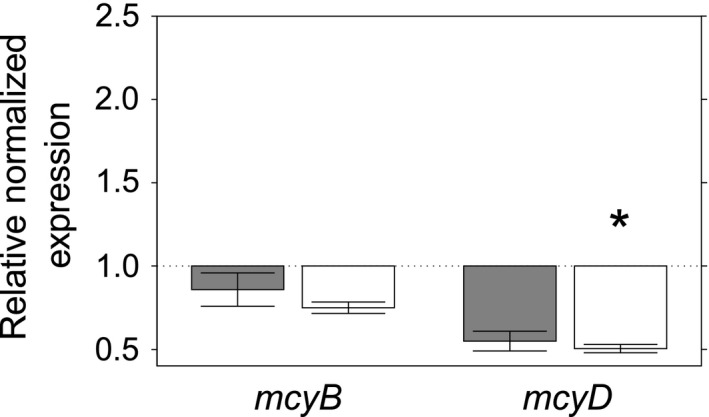
Relative normalized *mcyB* (left) and *mcyD* (right) mRNA expression in *Microcystis aeruginosa*. Error bars show standard error of the mean. *Statistically significant difference from control treatment, *p* ≤ .05. *Microcystis aeruginosa* cocultivated with 10^3^ cells/ml (gray) or 10^4^ cells/ml (white) *Ochromonas danica* for 48 hr. The control treatment, Ochromonas medium, was used for normalization. *Ochromonas danica* concentrations chosen were 10^3^ or 10^4^ cells/ml, as used in previous studies (Guo & Song, 2010; Van Donk et al., 2009)

Cocultivation of *M. aeruginosa* with *O. danica* did not increase the transcription levels of microcystin biosynthesis genes. In the contrary, *mcyD* transcription significantly decreased in the presence of *O. danica* (Figure 2). For *mcyD* transcripts, the mean fold‐change values decreased significantly for the cultures with 10^4^ cells/ml *O. danica* (*p *= .049) but not for 10^3^ cells/ml *O. danica* (*p *= .078). Transcription of *mcyB* was also decreased, but the difference was not significant (Table S1). These results show that the eukaryotic grazer *O. danica* does not induce the upregulation of microcystin biosynthesis genes but does even cause downregulation of relevant genes at the concentrations tested here. Therefore, the hypothesis that microcystin constitutes an inducible defense mechanism against unicellular grazers was not confirmed. Our results rather support the findings of others who found no evidence that microcystin acts as a defense against mixotrophic flagellates (Wilken et al., 2010). The possible role of microcystin as an infochemical has been discussed previously (Schatz et al., 2007), and using microcystin as an intercellular signal in the event of grazing pressure could potentially benefit the *M. aeruginosa* population. In light of this discussion, our findings are all the more surprising. However, we cannot exclude the possibility that another molecule serves such a signaling function.

In our study, *O. danica* did react to the presence of *M. aeruginosa* with a behavioral change like the one described by Pfandl, Posch, and Boenigk (2004). When no prey *M. aeruginosa* was present, the chrysophyte remained highly motile, moving around rapidly and frequently (Video S1). In the presence of *M. aeruginosa* cells, however, *O. danica* was mostly attached to the substratum and created a current with its flagella to capture prey bacteria (Video S2). The pivotal assumption that *O. danica* SAG933‐7 does prey on *M. aeruginosa* SAG14.85 was verified by microscopic observation of engulfed *M. aeruginosa* cells (Figure S1), which is in agreement with previous studies (Van Donk et al., 2009; Wilken et al., 2010).

Flagellates are thought to be responsible for more bacterial mortality than phages (Bettarel et al., 2003). This, and the fact that we did not observe an increase in toxin biosynthesis gene transcription in the presence of *O. danica*, suggests that the use of hetero‐ or mixotrophic flagellates is an interesting avenue to explore in the pursuit of controlling toxic cyanobacterial blooms.

### 3.3 Effects of the virioplankton size fraction

#### 3.3.1 Effects of the virioplankton size fraction from Lake Bergknappweiher before and during algal bloom

**Figure 3:**
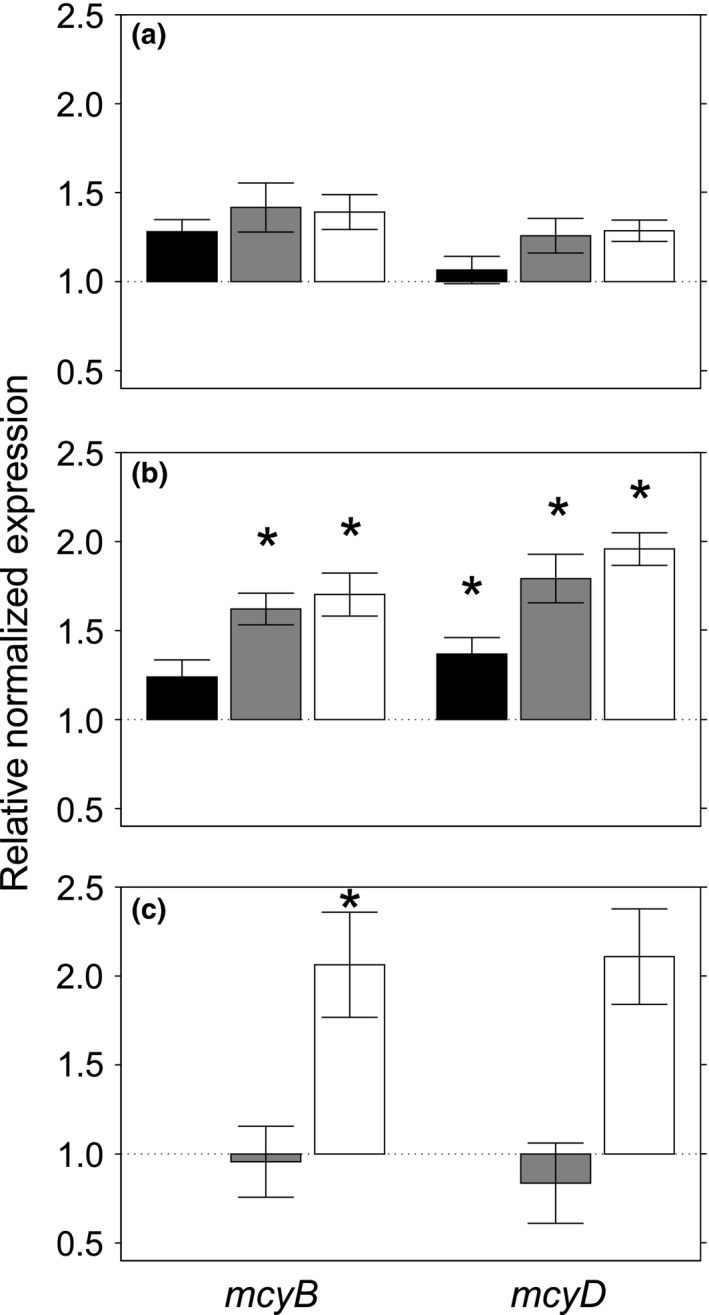
Relative normalized *mcyB* (left) and *mcyD* (right) mRNA expression in *Microcystis aeruginosa*. Error bars show standard error of the mean. *Statistically significant difference from control treatment, *p* ≤ .05. The control treatment, BG‐11 medium, was used for normalization. (a + b) Cocultivation of *M. aeruginosa* with autoclaved filtrate (black), 0.2 μm filtrate (gray), and particle concentrate (white) from Lake Bergknappweiher sampled before (a) and during (b) a cyanobacteria bloom for 48 hr. (c) Cocultivation of *M. aeruginosa* with 0.2 μm filtrate (gray) and particle concentrate (white) from Lake Klostersee sampled during a cyanobacteria bloom for 48 hr

Cultivation with 0.2 μm filtrate or particle concentrate from nonblooming Lake Bergknappweiher did not increase the transcription of *mcyB* or *mcyD* significantly (Figure 3a, Table S1).

**Figure 4:**
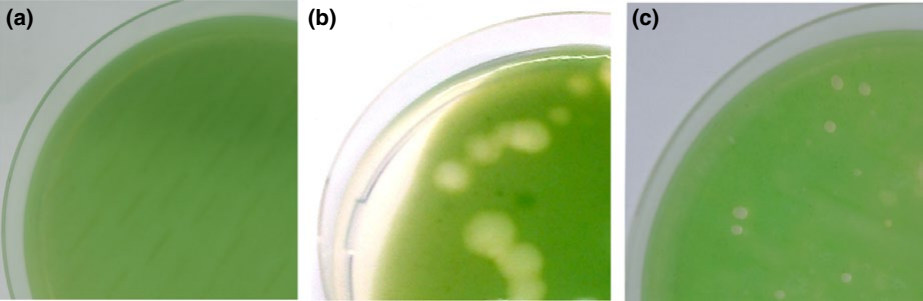
Plaque assay with *Microcystis aeruginosa* lawn. Water samples were collected on (a) 27 July 2015 from Lake Bergknappweiher when no cyanobacteria bloom was present, (b) 27 August 2015 from Lake Bergknappweiher with a cyanobacteria bloom present, and (c) on 25 June 2015 from Lake Klostersee with cyanobacteria bloom present. (b + c) Plaques on cyanobacteria lawn indicate presence of bacteriophages capable of infecting and lysing *M. aeruginosa*

However, 0.2 μm filtrate and particle concentrate from Lake Bergknappweiher sampled during an algal bloom did cause significant upregulation of microcystin biosynthesis genes in *M. aeruginosa* (Figure 3b). The cyanobacterial bloom was characterized by macroscopically visible phytoplankton aggregates on the water surface and a mass development of *Microcystis*‐like and *Dolichospermum*‐like cells. The *mcyB* mean fold‐change values of *M. aeruginosa* cultures exposed to 0.2 μm filtrate (*p *= .003) or particle concentrate (*p *= 0.002) differed significantly from that of controls. However, there was no significant change in cultures exposed to autoclaved filtrate (*p *= 0.240). Similarly, a statistically significant increase in *mcyD* transcripts of *M. aeruginosa* cultures exposed to 0.2 μm filtrate (*p* < .001) or particle concentrate (*p* < .001) was observed. For *mcyD*, even autoclaved filtrate caused a statistically significant rise in transcription (*p *= .020). These results show that *mcyB* and *mcyD* mRNA increased in *M. aeruginosa* cocultivated with the virioplankton size fraction from Lake Bergknappweiher water taken during an algal bloom. The different results for 0.2 μm filtrate from blooming and nonblooming water likely reflect higher concentrations of the effective components in the bloom samples (Figure 4a + b).

The presence of humic acid in Lake Bergknappweiher water was taken into account in a separate experiment. The change in water color due to the presence of humic acid, at two different concentrations, did not alter the transcription of microcystin biosynthesis genes significantly (Figure S2 and S3). The results show that the humic acid preparation tested in this study does not influence the transcription of selected toxicity genes and suggest that the observed changes in gene transcription after treatment with 0.2 μm filtrate and particle concentrate are not caused by humic acid in the water. Whether the chemical composition of the humic acid in Lake Bergknappweiher water influenced results, is less clear and needs to be subject of further study.

#### 3.3.2 Effects of the virioplankton size fraction from Lake Klostersee

Particle concentrate but not 0.2 μm filtrate from Lake Klostersee sampled during an algal bloom increased the transcription of microcystin biosynthesis genes (Figure 3c). The cyanobacterial bloom was characterized macroscopically by visible phytoplankton aggregates on the water surface and a mass development of *Microcystis*‐like cells. Mean *mcyB* fold‐change values of *M. aeruginosa* cultures exposed to particle concentrate differed significantly from those exposed to 0.2 μm filtrate and control cultures (*p *= .031). Mean *mcyD* fold‐change values in cultures exposed to particle concentrate differed significantly from those exposed to 0.2 μm filtrate (*p *= .049) but not control cultures (*p *= .064). These results show that microcystin synthetase gene transcription in *M. aeruginosa* increased when the cyanobacterium came into contact with small molecules and concentrated particles from water taken during an algal bloom in Lake Klostersee.

#### 3.3.3 Effective components in the virioplankton size fraction

There are several possible explanations for the observed effects caused by the virioplankton size fraction from environmental samples. One potential explanation is the presence of cyanophages in the environmental samples. We could demonstrate the presence of plaque‐forming units capable to infect *M. aeruginosa* in 0.2 μm filtrate from algal blooms (Figure 4b + c), and we found no such plaque‐forming units in the nonbloom water sample from Lake Bergknappweiher (Figure 4a). This presence of cyanophages is also reflected in a transcript upregulation when cocultivating *M. aeruginosa* with the virioplankton size fraction from bloom samples (Figure 3b + c). A further cue suggesting cyanophages as causative agents is the fact that the particle concentrate caused a higher increase in gene transcription than the 0.2 μm filtrate (Figure 3b + c). Viral particles are concentrated in the process of obtaining the particle concentrate unlike other small soluble compounds. Co‐occurring cyanophages might cause increased microcystin expression as defense against parasites. As microcystin is mostly located inside the cell (Park et al., 1998; Young, Thomson, Metcalf, Lucocq, & Codd, 2005), a defense function against intracellular pathogens such as bacteriophages is plausible. Microcystin and other secondary metabolites have been shown to reduce the virulence of parasitic fungi to the cyanobacterium *Planktothrix* (Rohrlack, Christiansen, & Kurmayer, 2013). The observation that autoclaved filtrate increased *mcyD* transcription in some cases (Figure 3b) seems to contradict this proposition. However, inactivated virus particles have been shown to cause infection‐independent transcriptional responses in the host cell (Huipao et al., 2017).

In addition, infochemicals or secondary metabolites produced by competing species may account for the observed effects on gene transcription, especially when considering the virioplankton size fraction from Lake Bergknappweiher. Such molecules are most likely present in larger amounts during algal blooms, which explains the difference in effects caused by the bloom and nonbloom virioplankton size fraction (Figure 3a + b). Additionally, the fact that autoclaved filtrate from an algal bloom caused an increase in *mcyD* transcripts (Figure 3b) points toward small heat‐stable metabolites as causative agent rather than heat‐sensitive bacteriophages. This explanation is strongly supported by the results reported here for the cocultivation of *M. aeruginosa* with spent medium from *S. elongatus* where soluble compounds caused an upregulation of *mcyB* transcripts (Figure 1).

Regardless of whether cyanophages or metabolites from other bacteria or a combination of both cause the observed effects on toxin gene transcription, our results show that those mechanisms are probably relevant not only for laboratory but also field conditions.

## 4 CONCLUSION

For the first time, the effect of a set of different microorganisms and the virioplankton size fraction from environmental water samples on *M. aeruginosa* was determined in a gene expression study. This study reveals the evident effects that co‐occurring microorganisms and the virioplankton size fraction have on the transcription of the microcystin biosynthesis genes *mcyB* and *mcyD* under laboratory conditions. While the virioplankton size fraction or the picocyanobacterium *S. elongatus* resulted in increased *mcyB* and *mcyD* transcription, the presence of the mixotrophic gazer *O. danica* resulted in decreased or unchanged transcription levels of those genes. Based on our observations, we do not deem cyanobacteria suitable tools for the biological control of toxic cyanobacterial algal blooms. Our findings highlight the complexity of interspecies interactions on the molecular level, and more research is needed to identify the key biological drivers in the expression of microcystin biosynthesis genes and microcystin synthesis. This study can serve as a starting point for future considerations in research and bioremediation.

## CONFLICT OF INTEREST

None declared.
