# Supplementary material for: Influence of cyanobacteria, mixotrophic flagellates, and virioplankton size fraction on transcription of microcystin synthesis genes in the toxic cyanobacterium Microcystis aeruginosa
Source: Microbiologyopen. 2017 Sep 25;7(1):e00538. doi: 10.1002/mbo3.538 (PMC5822348; doi:10.1002/mbo3.538)
Supplement: Supplementary file 1 [file MBO3-7-na-s001.docx]

Supplementary Information

**Influence of cyanobacteria, mixotrophic flagellates, and virioplankton size fraction on transcription of microcystin synthesis genes in the toxic cyanobacterium *Microcystis aeruginosa***

**Pia I. Scherer^*^, Carolin Absmeier, Maria Urban, Uta Raeder, Juergen Geist, Katrin Zwirglmaier**

*** Correspondence:** Pia I. Scherer: pia.scherer@tum.de

# 1. Supplementary Figures


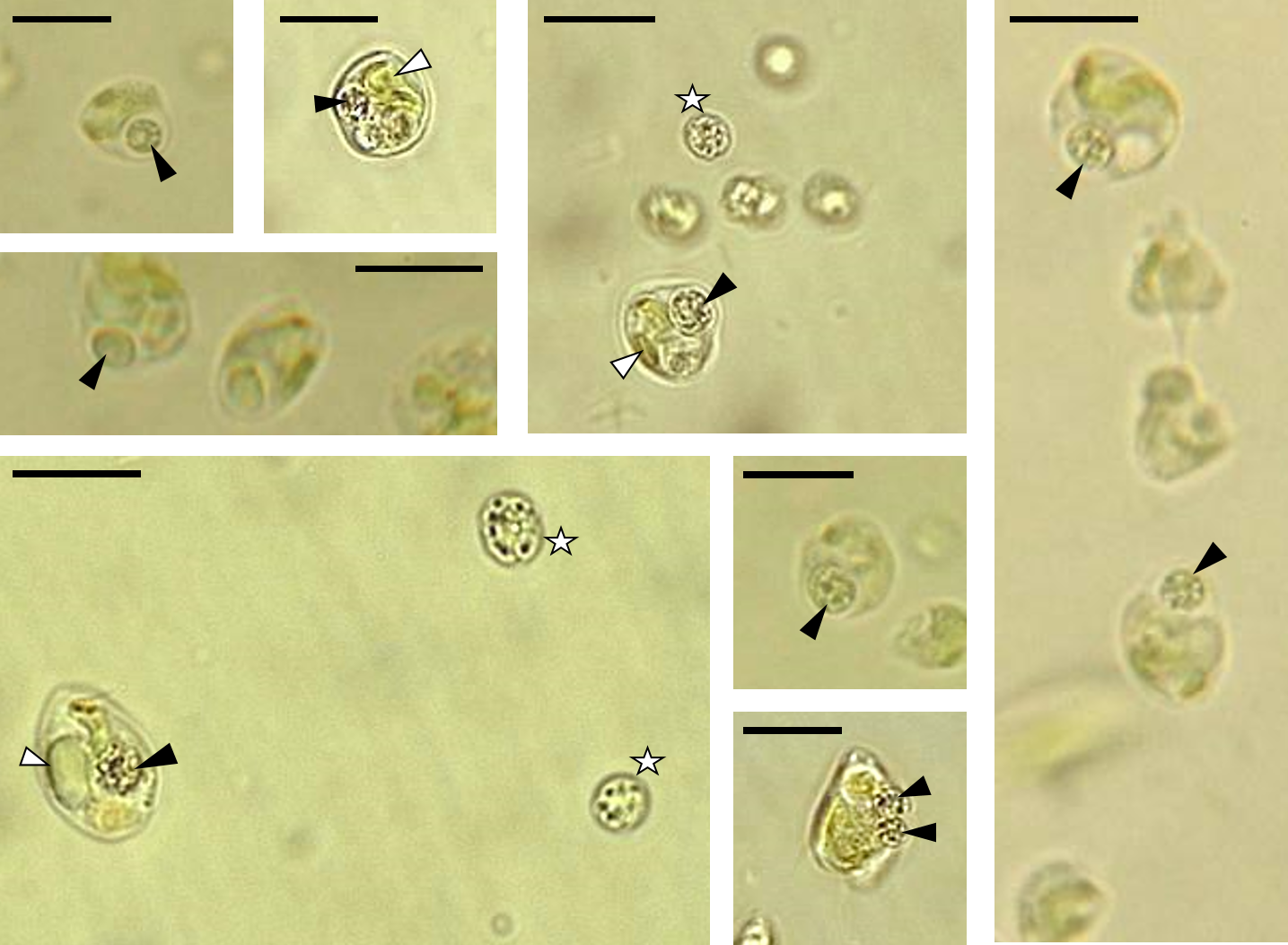


**Figure S1**. *O. danica* feeding on *M. aeruginosa*. Black arrowheads indicate engulfed *M. aeruginosa* cell within *O. danica* cell. White arrowheads indicate clearly distinguishable *O. danica* chloroplast. Stars indicate *M. aeruginosa* cells that are not engulfed. Bar = 10 µm.


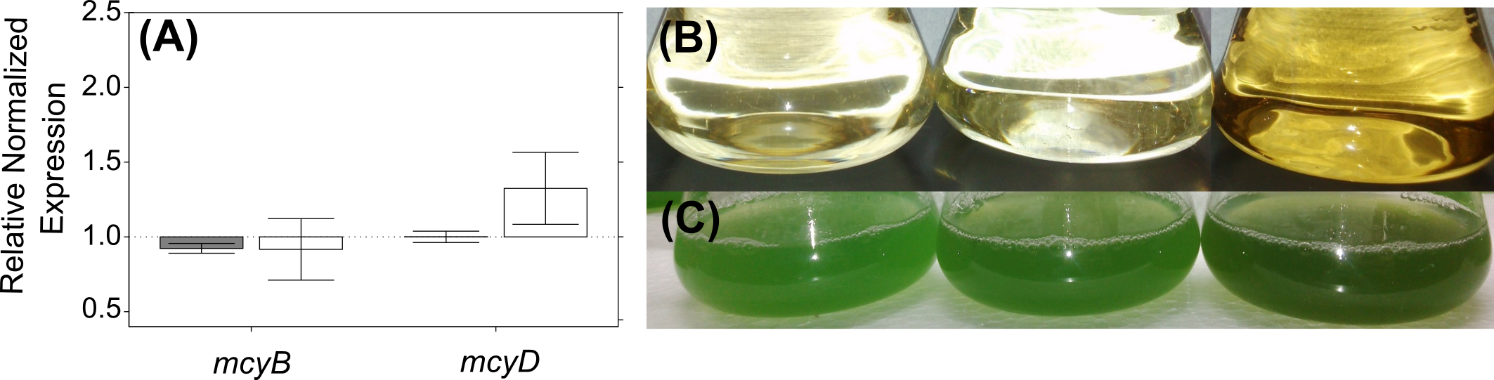


**Figure S2**. (A) Relative normalized *mcyB* (left) and *mcyD* (right) expression in *M. aeruginosa*. Error bars show standard error of the mean. Co-cultivation of *M. aeruginosa* with humic acid preparation similar to that from Lake Bergknappweiher (grey) or a 2.5-times more concentrated humic acid preparation (white). The control treatment, BG-11 medium, was used for normalization. Neither the humic acid preparation similar to the one in Lake Bergknappweiher nor the 2.5-times more concentrated humic acid preparation caused a significant mean fold-change in *mcyB* (*p*, 0.455) and *mcyD* (*p*, 0.058) expression compared to the control treatment. (B) From left to right: BG-11, humic acid preparation similar to that from Lake Bergknappweiher, and a 2.5-times more concentrated humic acid preparation. (C) From left to right: *M. aeruginosa* cultures mixed with BG-11, humic acid preparation similar to that from Lake Bergknappweiher, and a 2.5-times more concentrated humic acid preparation.


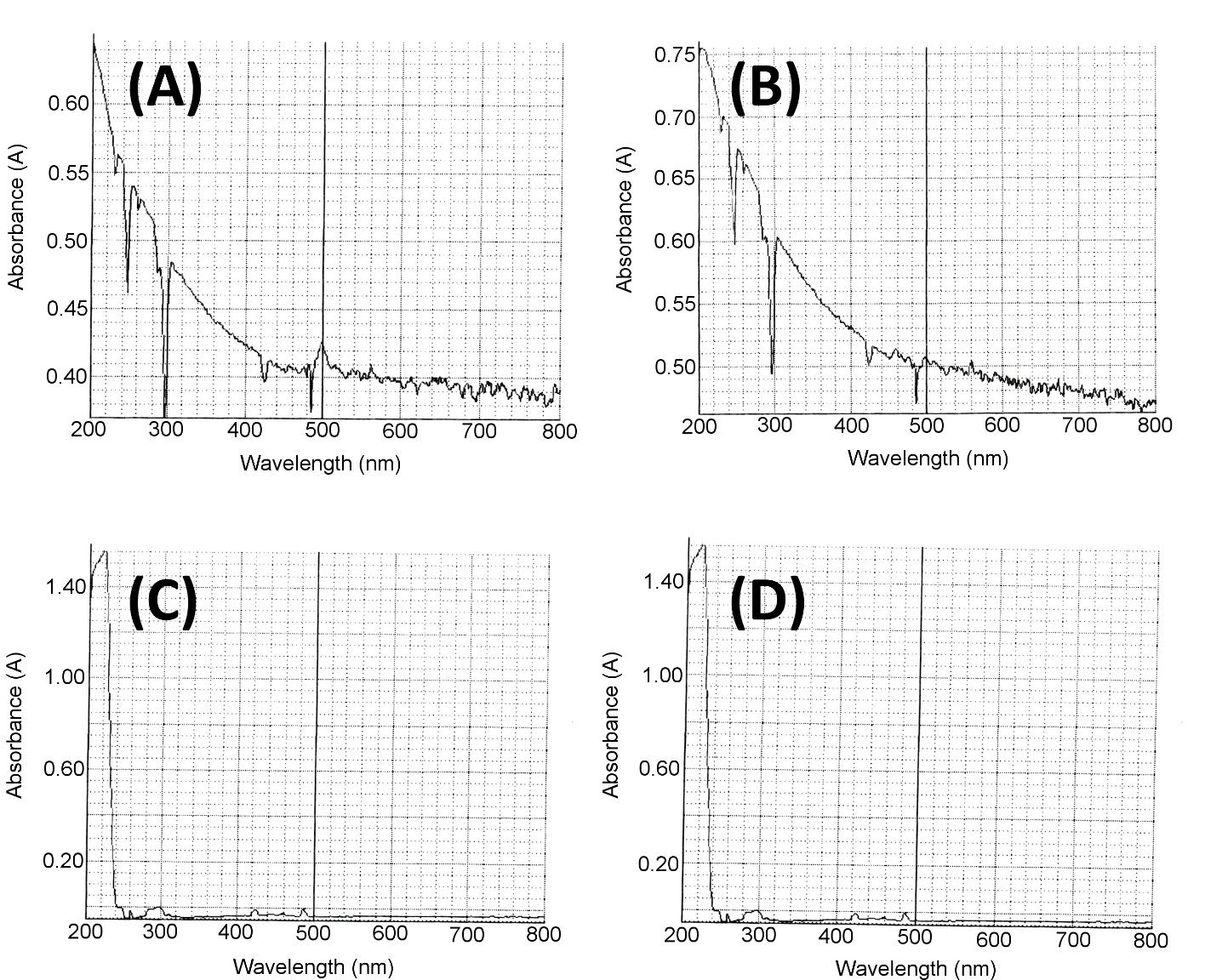


**Figure S3**. Absorption spectra between 200 nm and 800 nm wavelength. (A) Water from Lake Bergknappweiher before bloom, 0.2 µm filtered. (B) Water from Lake Bergknappweiher during bloom, 0.2 µm filtered. (C) Humic acid preparation, visually similar to Lake Bergknappweiher water. (D) Humic acid preparation, 2.5-fold more concentrated than in (C).

# 2. Supplementary Tables

|  |  | ANOVA | | | |  | Tukey’s post hoc  test | | |
| --- | --- | --- | --- | --- | --- | --- | --- | --- | --- |
|  |  | df between groups | df within groups | *F* | *p* |  | SEM | Mean fold change | Tukey’s post hoc  *p* |
| Cyanobacteria | *mcyB*  *mcyD* | 2  2 | 15  15 | 12.68  5.02 | <0.001  0.021 | control | 0.08  0.07 | 1.00  1.00 |  |
|  |  |  |  |  |  | spent medium | 0.09  0.11 | 1.70  1.59 | 0.003  0.064 |
|  |  |  |  |  |  | cells | 0.08  0.08 | 1.76  1.73 | 0.001  0.025 |
| Mixotrophic Flagellates | *mcyB mcyD* | 2  2 | 15  14 | 1.20  4.40 | 0.329  0.033 | control | 0.22  0.15 | 1.00  1.00 |  |
|  |  |  |  |  |  | 10^3^ cells/mL | 0.10  0.06 | 0.86  0.55 | ND  0.078 |
|  |  |  |  |  |  | 10^4^ cells/mL | 0.03  0.02 | 0.75  0.50 | ND  0.049 |
| Lake Bergknappweiher virioplankton size fraction  (no bloom) | *mcyB mcyD* | 3  3 | 8  8 | 4.61  3.02 | 0.037  0.094 | control | 0.07  0.06 | 1.00  1.00 |  |
|  |  |  |  |  |  | autoclaved phages | 0.07  0.08 | 1.28  1.07 | 0.572  ND |
|  |  |  |  |  |  | env. phages | 0.14  0.10 | 1.42  1.26 | 0.052  ND |
|  |  |  |  |  |  | phage concentrate | 0.10  0.06 | 1.39  1.29 | 0.062  ND |
| Lake Bergknappweiher virioplankton size fraction  (algal bloom) | *mcyB mcyD* | 3  3 | 8  8 | 16.25  41.48 | <0.001  <0.001 | control | 0.10  0.09 | 1.00  1.00 |  |
|  |  |  |  |  |  | autoclaved phages | 0.10  0.09 | 1.24  1.37 | 0.240  0.020 |
|  |  |  |  |  |  | env. phages | 0.09  0.14 | 1.62  1.79 | 0.003  <0.001 |
|  |  |  |  |  |  | phage concentrate | 0.12  0.09 | 1.70  1.96 | 0.002  <0.001 |
| Lake Klostersee virioplankton size fraction  (algal bloom) | *mcyB mcyD* | 2  2 | 7  7 | 8.25  5.85 | 0.026  0.049 | control | 0.13  0.10 | 1.00  1.00 |  |
|  |  |  |  |  |  | env. phages | 0.20  0.23 | 0.96  0.84 | 0.998  0.967 |
|  |  |  |  |  |  | phage concentrate | 0.30  0.27 | 2.06  2.11 | 0.031  0.064 |
| Humic acid preparation | *mcyB mcyD* | 2  2 | 7  7 | 0.93  5.29 | 0.455  0.058 | control | 0.05  0.06 | 1.00  1.00 |  |
|  |  |  |  |  |  | humic acid 1x | 0.03  0.04 | 0.92  1.00 | ND  ND |
|  |  |  |  |  |  | humic acid 2.5x | 0.21  0.24 | 0.92  1.32 | ND  ND |

**Table S1**. Detailed statistics results. ANOVA: df: degrees of freedom, F: F-value, p: p-value; Tukey’s post hoc test: SEM: standard error of the mean, ND: not determined, upper number indicating result of statistic test for *mcyB*, lower number indicating result of statistic test for *mcyD*.

| Experiment | Sample | OD_730_ |
| --- | --- | --- |
| Lake Bergknappweiher virioplankton size fraction (no bloom) | control, replicate 1 | 0.503 |
|  | control, replicate 2 | 0.567 |
|  | control, replicate 3 | 0.514 |
|  | autoclaved filtrate, replicate 1 | 0.606 |
|  | autoclaved filtrate, replicate 2 | 0.681 |
|  | autoclaved filtrate, replicate 3 | 0.591 |
|  | 0.2 µm filtrate, replicate 1 | 0.621 |
|  | 0.2 µm filtrate, replicate 2 | 0.677 |
|  | 0.2 µm filtrate, replicate 3 | 0.631 |
|  | particle concentrate, replicate 1 | 0.540 |
|  | particle concentrate, replicate 2 | 0.712 |
|  | particle concentrate, replicate 3 | 0.624 |
| Lake Bergknappweiher virioplankton size fraction (algal bloom) | control, replicate 1 | 0.499 |
|  | control, replicate 2 | 0.454 |
|  | control, replicate 3 | 0.414 |
|  | autoclaved filtrate, replicate 1 | 0.522 |
|  | autoclaved filtrate, replicate 2 | 0.573 |
|  | autoclaved filtrate, replicate 3 | 0.544 |
|  | 0.2 µm filtrate, replicate 1 | 0.476 |
|  | 0.2 µm filtrate, replicate 2 | 0.557 |
|  | 0.2 µm filtrate, replicate 3 | 0.447 |
|  | particle concentrate, replicate 1 | 0.490 |
|  | particle concentrate, replicate 2 | 0.463 |
|  | particle concentrate, replicate 3 | 0.498 |
| Lake Klostersee virioplankton size fraction  (algal bloom) | control, replicate 1 | 0.345 |
|  | control, replicate 2 | 0.404 |
|  | control, replicate 3 | 0.310 |
|  | 0.2 µm filtrate, replicate 1 | 0.451 |
|  | 0.2 µm filtrate, replicate 2 | 0.49 |
|  | 0.2 µm filtrate, replicate 3 | 0.476 |
|  | particle concentrate, replicate 1 | 0.546 |
|  | particle concentrate, replicate 2 | 0.524 |
|  | particle concentrate, replicate 3 | 0.478 |
| Humic acid preparation | control, replicate 1 | 0.614 |
|  | control, replicate 2 | 0.630 |
|  | control, replicate 3 | 0.631 |
|  | humic acid 1x, replicate 1 | 0.669 |
|  | humic acid 1x, replicate 2 | 0.663 |
|  | humic acid 1x, replicate 3 | 0.654 |
|  | humic acid 2.5x, replicate 1 | 0.652 |
|  | humic acid 2.5x, replicate 2 | 0.653 |
|  | humic acid 2.5x, replicate 3 | 0.672 |

**Table S2**: Optical densities at wavelength 730 nm (OD_730_) after co-cultivation with virioplankton size fraction or humic acid.

# 3. Supplementary Videos

**Video S1**. *O. danica* grown in Ochromonas medium and without prey bacteria. *O. danica* is highly motile in the absence of prey bacteria.

**Video S2**. *O. danica* grown in Ochromonas medium and co-cultivated with *M. aeruginosa* for two days. *O. danica* attached to the substratum and created a medium current with its flagella in which prey bacteria (*M. aeruginosa*) spin.
